# Ingrowing Toe-Nail

**Published:** 1890-10

**Authors:** 


### INGROWING TOE-NAIL.

This subject may seem trivial to the surgeon or physician, who goes in for the so-called capital operations, but it is of considerable interest to him who seeks to relieve the suffering of humanity in any form which it may take.

Dr. Joseph Amiard, of Paris, has an exhaustive treatise upon this subject, in Wood's *Medical and Surgical Monographs* for August. He first calls to mind some obscure points in the etiology of the affection. With a few exceptions the time of occurrence has been observed to be limited between the ages of fourteen and thirty years respectively. While some authors attribute the disease to the nail itself or the surrounding parts, most surgeons attribute it to the shoes. Besides the mechanical action of the shoes, which is quoted as described by many writers, there are cases which are so-called idiopathic; as, "in convalescence from acute diseases; in typhoid fever, of which Reclus reports a case with considerable fungoid growth; in the course of chronic diseases after considerable debility; in syphilis, which acts through its cutaneous eruptions; in peri-onyxis, scrofula, diabetes (Verneuil); in individuals of a lymphatic temperament; and Gosselin has previously noted the influence of the general condition of the body, in the absence of any occasional cause." Changes referable chiefly to the skin and epidermal appendages, in which latter the nail is included, may occur after organic lesions, as aneurism, but especially after central and peripheral nerve lesions. After reviewing these causes the author says they are, taken separately, either too exclusive or too absolute. Narrowness of shoes should render the trouble much more frequent "in women, who, from vanity, imprison their feet in narrow shoes," but statistics do not sustain this view. In exceptional cases the cause cannot be ascertained. (Is it not possible that certain cases may be hereditary, or even congenital ?—Ed.)

After a full review of the "palliative" treatment, which includes the mechanical applications and operations which are generally recommended, he concludes that none of these procedures absolutely prevent a recurrence. He says we must not only remove the nail, but also the nail-producing tissues. The surgeon must remove the exuberant growths, the ingrowing nail, and the matrix, the persistence of which is the cause of so many failures. Anatomists had not fully settled the question of the source of nail-growth, every portion of the bed, edges and root being credited with a share in reproduction. He says M. Quènu definitely settled this question, in 1887, by experiment. He twisted off the nail, removing the entire semi-lunar surface without touching the anterior part of the subungual dermis. If all the cells of the dermis could re-form the nail, the anterior part at least should re-form independently of the "root"; if the cells of the semi-lunar part could alone reproduce nail, the nail would not form again after the above operation. It did not form again. At the anterior undisturbed part only a few horny lamellæ were formed, which did not cohere to form a membrane, or nail-substance. Acting upon this, the removal of the semi-lunar surface will be radical and successful, preventing relapse.

